# Dietary nutrient allocation to somatic tissue synthesis in emerging subimago freshwater mayfly *Ephemera danica*

**DOI:** 10.1186/s12898-018-0213-9

**Published:** 2018-12-14

**Authors:** Elizabeth Yohannes, Karl-Otto Rothhaupt

**Affiliations:** Limnological Institute, University of Konstanz, Mainaustrasse 252, 78464 Constance, Germany

**Keywords:** δ^13^C, δ^15^N, Insect, Nutrient, Somatic tissues, Lake Constance

## Abstract

**Background:**

The relative importance of nutrients derived from different sources for tissue synthesis is crucial for predicting a species responds to changes in food availability. The ecological and physiological strategies that govern the incorporation and routing of nutrients for reproduction are often well understood. However, the role and adaptive value of both species and individual variation during early life-stage remain elusive. In freshwater systems, dietary nutrient allocation to somatic tissue should be favoured when dietary source peaks and resource limitation may hinder flexible resource allocation. We used carbon and nitrogen stable isotopes (δ^13^C and δ^15^N) to examine metabolic nutrient routing and resource allocation from four dietary sources used to biosynthesize three somatic tissues of emerging subimago *Ephemera danica*. Aquatic emerging insects, such as the mayfly *E. danica,* are well suited for such studies. This is because, while burrowing nymph phase is a detritivores feeders with several early life-stages of metamorphosis, adult insects do not feed during this period but do utilize energy.

**Results:**

Constructed models to predict percent proportional contribution of source to tissue showed that terrestrial detritus was the dominant nutrient source for abdomen, head and wing with mean values of 57%, 65% and 73%, respectively. There was evidence for differential resource allocation, as insect partitioned periphyton and sediment (but also seston) elements for tissue synthesis. Utilizing individual-specimen based relationship in isotope value; we derived tissue specific isotopic niche estimates, for the different tissue-source combinations.

**Conclusions:**

Results indicate that tissue selection is crucial for isotopic ecological measurements in arthropods. Mayfly has long been used as bio-indicator of freshwater ecosystems and their larvae show rapid response to environmental changes. In light of the recent evidence of drastic reduction in flying insect mass in Germany, developing a system using isotopic tools to trace nutrient flow in this important taxon will assist conservation and management efforts.

## Background

The relative importance of nutrients derived from different sources for tissue synthesis is crucial for predicting how species respond to changes in food availability through their life cycle.

Resource acquisition and allocation across life cycle stages to support develop, somatic tissue synthesis are central in ecology and evolution. In aquatic emerging insects, definite developmental stages are strongly linked to feeding and fasting (non-feeding) life cycle phases that can influence each other. Typically, the burrowing larval stage feeds, while adult individuals do not feed. Thus adult insects rely on energy acquired during larval stages to maintain the major energy budget during adulthood to meet metabolism and reproductive effort [1, 2].

Most larval insects metamorphose from aquatic larvae to winged emerging adults, and larvae must acquire sufficient nutrients to fuel the developmental stage and to support the

late larval and early adult periods. Recent surveys indicate that that metamorphosis affects the nutrient values due to ontogenetic shift in resource utilization use during metamorphosis [3–5]. However, our understanding regarding larval energy stores, resource mobilization and released for use during different developmental stages, and for somatic maintenance is still at its lower stage, although the specific methods (such isotopic approach) have well advanced over a long period and isotopes have been used to reveal the trophic ecology of insects over the last 30 years (reviewed by [6–8]).

Aquatic insects with burrowing larvae, such as the mayfly *Ephemera danica,* are classical detritivores feeders, appearing to selectively feed on microbe-colonized detritus [9]. Moreover, bacterial-based diet provides a substantial part of the vital nitrogen requirement of these species [10, 11]. Elsewhere, nearshore burrowing macroinvertebrates have been shown to sustain diets that included carbon derived from biogenic methane attributed to assimilation of methanotrophic bacteria [12].

Optimal nutrient allocation to somatic tissue synthesis might depend upon internal resource demands and external environmental conditions [13]. Since resource limitation may hamper plasticity in nutrient allocation, individuals may show temporal shift in resource use for tissue processing. Dietary nutrient allocation to somatic tissue synthesis (such as abdomen, head or wing) in emerging mayfly are expected to exhibit greater variation in nutrient routing from which they are synthesized due to their relatively larger difference in mass contribution and growth [8]. Certainly, the degree to which the isotopic signature of the tissue reflects the diet of the resource will depend on the time since tissue synthesis and nutrient turnover rates [14–16]. Unfortunately, tissue specific isotopic turnover rates for carbon and nitrogen in abdomen, head, wing or even whole body for *Ephemera danica* are not available.

Emerging mayfly tends to involve enormous numbers of individuals within a few meters of the aquatic area (e.g. Lake), providing ample opportunity to transport stored nutrient to terrestrial interface. This also facilitates the collection of a large number of insects within a given sampling period and minimizes the effect of emergence time on isotope data interpretation. Moreover, the abundance of naturally occurring stable isotopes in the tissues of an emerging mayfly offers many advantages over other tools used for determining diet composition and resource allocation. This is mainly due to the fact that conventional approaches for investigating consumers’ nutrient sources, such as gut-content analysis and visual observation are practically impossible.

Finally, due to the insects’ small size, scientists typically apply stable isotope composition of insects by using either entire individuals, or simply a single tissue part, such as parts of wings [14, 17]. Here, we examined the stable isotope values of individual emerging insects and their isotopic signal in three specific tissues in relation to four of their potential primary dietary (nutrient sources). We used these data primarily in a Bayesian mixing model to estimate the relative carbon (C) and nitrogen (N) contributions for somatic tissue synthesis. Our objectives included to shed light on inter-individual variation in dietary use (and nutrient routing) to somatic tissue allocation in the species.

## Methods

We explored stable isotope values (carbon, δ^13^C and nitrogen, δ^15^N) in abdomen, head and wing tissues of emerging subimago mayfly from Lake Constance (Germany). We then examined the patterns of individual variation in the use of nutrient sources from local diet originating from periphyton, seston, sediment and terrestrial detritus. The mayfly is especially well suited for the purpose since the immature and generally aquatic nymph phase achieves several stages of indirect metamorphosis (thus tissue growth stored dietary resources) while the adults don’t feed yet utilize energy [3].

In order to investigate whether individuals differ in the degree to which they utilize dietary resources for different somatic tissue syntheses, we considered individual insects of equivalent size (ca. 2 mg dry weight) and compared the stable isotope values in three tissues (abdomen, head and wing). Although the detailed life-history patterns of the current Lake Constance ephemeropteran community is not well studied, a 6-year study from 1966 to 1972 reported 37 species in the region (including *E. danica*) of which 10 were considered to be rare and endemic [18].

### Sample collection

#### Mayfly *Ephemera danica*

Emergent *E. danica* were collected in a single sampling event in July 2011 in the littoral zone of Lake Constance (Egg, near the city of Konstanz, 47° 41′ 46″N; 9° 11′ 31″E) using a regular mesh net. Two sub-sets of insects were collected to explore

- Population-wide differences between tissue isotope and for source comparison
  We used δ^13^C and δ^15^N values obtained from a 141 individual emerging insect to examine metabolic nutrient routing and resource allocation from four dietary sources used to synthesize three somatic tissues of emerging insect. Briefly, from each emerging individual, a single tissue-section was collected [i.e. abdomen (N = 41), head (N = 53) or wing (N = 47)]. These values were applied to construct models and to predict percent proportional contribution of four dietary sources to three somatic tissue syntheses.
- Within-in individual differences between tissue isotope values (same subjects simultaneous sampling)
  This test was conducted to specifically compare abdomen, head and wing (simultaneous sampling) from each individual (individual specific pair-wise tissue isotopic comparisons). For this purpose, we dissected 17 adult individuals and simultaneous sampled three tissue specimen (abdomen, head and wing) from each emerging insect.

#### Sediment, seston, periphyton and terrestrial detritus

In order to compare tissue δ^13^C and δ^15^N values, we used isotope mixing model that contained four potential dietary components and applied a Bayesian modeling framework. For this, we collected sediment, seston, periphyton and terrestrial detritus from the same site as follow:

A surface sediment sample was obtained using a sled to collect material from the top 1–2 cm layer. Sediment samples were then sieved through a 250 μm sieve to retain fauna. The sediment that passed through the sieve was retained for analysis and sediment sample greater than 250 µm (including the fauna) was discarded. To obtain seston samples, lake water was sampled using a Ruttner sampler and a sub-sample of approximately 1 L was filtered through a pre-combusted glassfiber filter (Whatman GF/F). Periphyton was collected by gently removing growth from rocky substrates using a brush sampler. Additionally, samples of decomposing leaves and samples of terrestrial detritus < 2 mm were collected from the shore.

All samples were collected once per week over seven a week time prior to collecting insects (Egg, near the city of Konstanz, 47° 41′ 46″N; 9° 11′ 31″E). Seston, periphyton and detritus materials constitute dead particulate organic material (as opposed to dissolved organic material) that typically includes the bodies or fragments of dead organisms, decomposing and demineralize materials. These, presumably for a much larger number of elements, provided that sampled early enough prior to the emergency season, delineate resource provenance to tissue synthesis.

#### Stable isotope analyses

Lipids were extracted from all animal tissue samples (whole body tissue and each body parts: head, abdomen and wing) by soaking them in a 2:1 chloroform methanol solution for 48 h followed by rinsing using distilled water. Carbonate was removed from sediment, seston and periphyton samples of (5–7 mg) using 1 M HCl. Animals, bulk sediment (N = 8), periphyton (N = 8) and seston (N = 8) samples were then oven-dried (60 °C) and powdered, packed individually into tin capsules (ca. 0.8 mg) and analysed for elemental and stable isotope content at the University of Konstanz, stable isotope core facility, Germany.

Powdered sub-samples of approximately 0.8 mg were weighed to the nearest 0.001 mg in small tin cups, using a micro-analytical balance. Samples were then combusted in a vario Micro cube elemental analyzer (Elementar, Analysensysteme, Germany). The resulting CO_2_ and N_2_ were separated by gas chromatography and passed into the inlet of an Isoprime (Micromass, Manchester, UK) isotope ratio mass spectrometer (IRMS) for determination of ^13^C/^12^C and ^15^N/^14^N ratios. Measurements are reported in δ-notation (δ^13^C and δ^15^N) where notation in parts per thousand deviations (‰) relative to international standards for carbon (Pee Dee Belemnite, PDB) and nitrogen (atmospheric N_2_), according to the equation δ (‰) = 1000 × [(R_sample_)/(R_standard_) − 1]. Two sulfanilamides (Iso-prime internal standards) and two casein standards were used for every eight unknowns in sequence. Internal laboratory standards indicated measurement errors (SD) of ± 0.03‰ for δ^13^C, 0.12‰ for δ^15^N.

#### Data analysis

- Population-wide differences between multiple tissue isotope values and source comparison
  We used analysis of variance (ANOVA) to evaluate population-wide differences between tissue isotope values in δ^13^C or δ^15^N and to test whether the measured values among nutrient sources: δ^13^C periphyton (δ^13^C_periphyton_), seston (δ^13^C_seston_), sediment (δ^13^C_sediment_) and terrestrial detritus (δ^13^C_detritus_) or δ^15^N periphyton (δ^15^N_periphyton_), seston (δ^15^N_seston_) sediment (δ^15^N_sediment_) and terrestrial detritus (δ^15^N_detritus)_ are different. Where we found significant differences (at p < 0.05), we used Tukey’s honest significant difference (HSD) post hoc test to detect specific differences. We first applied normality test (Kolmogorov–Smirnov) and homogeneity of variances using Levene’s test. Although the equal variance test for tissue δ^15^N values failed (p = 0.01), we applied Tukey’s post hoc test since it is a relatively conservative for pairwise multiple comparison procedures.
- Within-in individual differences between tissue isotope values (same subjects simultaneous sampling)
  Repeated measure analysis of variance (RM-ANOVA) was carried out to test for mean individual differences between tissue δ^13^C values of abdomen (δ^13^C_abdomen_), head (δ^13^C_head_) and wing (δ^13^C_wing_) or δ^15^Nvalues of abdomen (δ^15^N_abdomen_), head (δ^15^N_head_) and wing (δ^15^N_wing_). With such design, the repeated-measure (independent variable) is the within-subjects factor that is tested for each insect subject at δ^13^C or δ^15^N (the dependent variable). A Bonferroni test, with a single pooled variance, was used to compare multiple pairwise comparisons of tissues based on each elemental ratio. Finally, we investigated the elemental relationship between paired tissue δ^13^C and δ^15^N isotopes values using ordinary least square linear regression and calculated the slope without prey items included.

#### Mixing models and coefficient of variation

We constructed mixing model to predict the proportional contribution of each of four nutrient sources (periphyton, seston, sediment and terrestrial detritus) to insect tissues in Lake Constance. To represent uncertainties in potential dietary items and diet–tissue discrimination factors, we used the MixSIAR mixing model programmed in R version 3.2.3 [19–21]. The model applies Markov chain Monte Carlo (MCMC) methods in a Bayesian framework to estimate the relative contribution of sources (potential dietary items) to a mixture (consumer tissue). It then provides probability density estimates for dietary-source proportions [22]. We used the general pattern of diet–tissue discrimination factors expected for consumers (+1.1‰ ± 0.2‰ for ^13^C and +3.4‰ ± 0.2‰ for δ^15^N).

#### Tissue-specific differences in niche indices

Isotopic diversity indices were measured for tissue samples [abdomen (N = 17), head (N = 17) and wing (N = 17), using the SIBER (Stable Isotope Bayesian Ellipses in R) package for R v.2.10.159 [23]. We calculated the standard ellipse area, SEA which is comparable to standard deviation (SD), and its corresponding SEA_C_ (SEA corrected for small sample size) to describe core aspects of tissue-specific niche [24, 25]. The SEA_C_ overlap between tissues was calculated for each tissue combination [24]. Tissue differences in SEA_C_ were calculated using Bayesian inference (SEA_B_) [24]. Finally, we estimated tissue-specific percentages of coefficient of variation for δ^13^C (CVc %) and for δ^15^N (CVn %), representing niche diversity of the basal resource and trophic diversity, respectively.

## Results

### Population-wide differences between tissue isotope values

#### Multiple tissue and source comparison

**Fig. 1:**
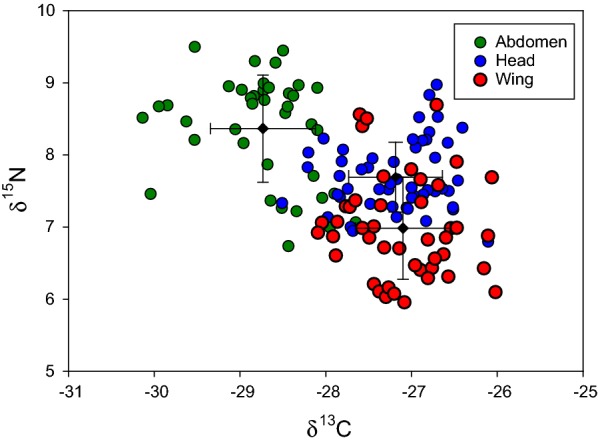
Stable isotope bi-plots illustrating the abdomen, head and wing isotope values of E*phemera danica*

**Fig. 2:**
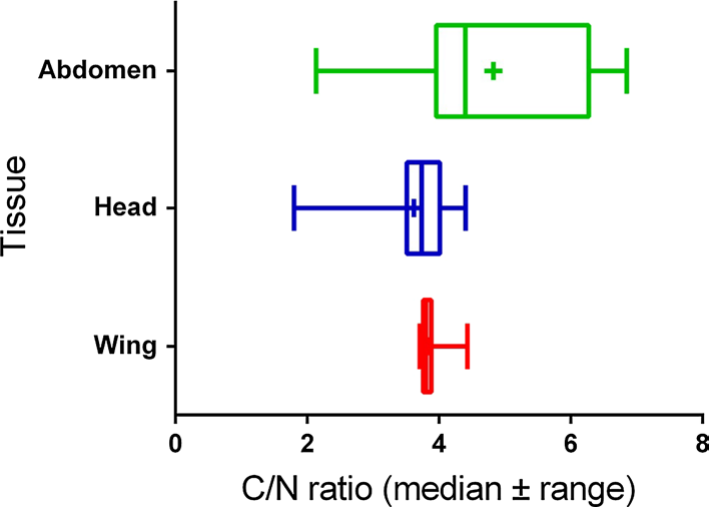
Carbon and nitrogen ratio in abdomen, head and wing of E*phemera danica*

A summary of carbon and nitrogen isotope values in source and tissue of mayfly *E. danica* is given in Table 1. Variance analysis depicted that there were significant differences in δ^13^C (F_[2,90]_ = 90.14, p < 0.0001) and δ^15^N (F_[2,90]_ = 55.617, p < 0.0001) between insect head, abdomen and wing tissues. Potential insect nutrient sources were also highly variable in δ^13^C (F _[3,26]_** = **67.68, p < 0.0001) and δ^15^N (F _[3,26]_ = 92.06, p < 0.0001). Insect tissues were generally δ^13^C-enriched and δ^15^N-depleted compared with nutrient sources, except relative to terrestrial detritus. Though δ^13^C_seston_, δ^13^C_sediment_ and δ^13^C_detritus_ were not statistically distinct from one another, due to their differences in δ^15^N, we held them as separate groups in the mixing model. Periphyton dietary sources were differentiated from seston and sediment but also from terrestrial detritus both by δ^13^C and δ^15^N values (Table 1). The δ^13^C_abdomen_ was differentiated from both δ^13^C_head_ and δ^13^C_wing_, and separation between all other tissues was achieved with δ^15^N values (δ^15^N_abdomen_, δ^15^N_head_ and δ^15^N_wing_, Table 1), as evidenced by their relative position in dual isotope space depicted in Fig. 1. Finally, the mean C/N mass ratio (± SD) of abdominal, head and wing tissues were 4.83 ± 1.37, 3.61 ± 0.65 and 3.84 ± 0.17, respectively (Fig. 2).

**Table 1:** Stable carbon and nitrogen isotope values in source and tissue of mayfly *Ephemera danica*

Source/Tissue	δ^13^C (‰) ± SD	δ^15^N (‰) ± SD
Abdomen	− 28.27 ± 1.12^a^	7.45 ± 0.58^a^
Head	− 27.27 ± 0.77^a^	7.37 ± 0.35^b^
Wing	− 27.53 ± 0.35^c^	6.90 ± 0.46^c^
Periphyton	− 22.56 ± 1.57^d^	15.65 ± 2.21^d^
Seston	− 29.19 ± 0.44^c^	10.83 ± 1.03^e^
Sediment	− 29.42 ± 0.63^c^	8.48 ± 0.86^c^
Terrestrial detritus	− 28.92 ± 0.98^c^	4.18 ± 0.97^g^

Samples sharing common letters within a column are not significantly different (following Tukey’s honest significant difference (HSD) post hoc test and ANOVA (F_[6,117]_ = 91.14, p < 0.0001 and F_[6,117]_ = 144.58, p < 0.0001) for δ^13^C and δ^15^N values, respectively)

### Mixed model

A summary of the proportional estimates of dietary sources for three tissue synthesis in mayfly *E. danica* is given in Table 2. Despite the overall dominance and potential of terrestrial detritus and sediment as the main elemental source for mayfly tissue formation (Table 2), we generally found overlap in estimated nutrient sources for tissues synthesized. Terrestrial detritus has the greatest mean contribution to the three tissue syntheses, particularly to wing tissue (ca. 74% proportional contribution). Seston was the least important for head and wing tissues (range of mean 3–5%). With the exception of periphyton, abdomen had correspondingly high organic resource contribution from terrestrial detritus, sediment and seston (ca. 57%, 25% and 16%, respectively).

**Table 2:** Proportion estimates of dietary sources (mixing model using both δ^13^C and δ^15^N), for three tissue synthesis in mayfly *Ephemera danica*

Source % (mean ± 95 CI)	Abdomen	Head	Wing
Periphyton	1.24 (0–3)	16.08 (14–19)	17.06 (14–20)
Seston	16.19 (2–30)	5.33 (0–12)	3.29 (0–8)
Sediment	25.48 (7–44)	13.099 (3–22)	5.67 (0–11)
Terrestrial detritus	57.09 (51–63)	65.55 (61–70)	73.98 (69–79)

*CrI* credible intervals. (Appendix Fig. 5)

### Within-in individual differences between tissue isotope values

#### Same subjects simultaneous tissue sampling

**Fig. 3:**
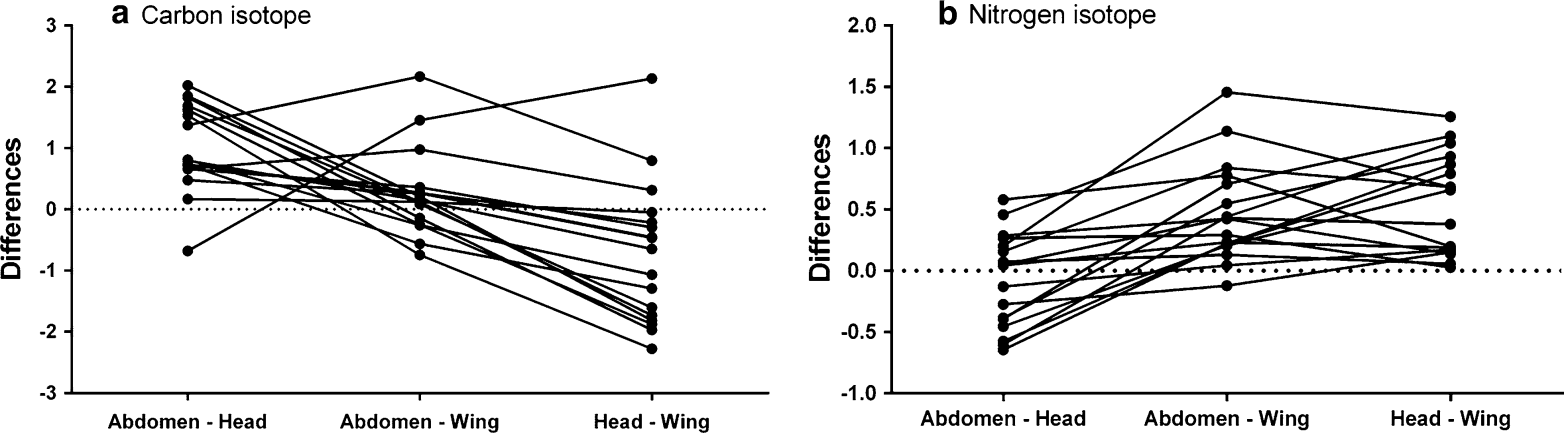
Differences in actual isotope signatures of simultaneously sampled abdomen, head and wing tissues of individual specimen

RM-ANOVA exhibited significant difference between individual insect carbon isotope values (F _**[**16,32]_** = **3.05, p = 0.0035) and nitrogen isotope values (F _[16,32]_** = **6.42, p < 0.0001). Bonferroni’s multiple comparisons test confirmed significant difference between δ^13^C_abdomen_ and δ^13^C_head_ (p < 0.001; Figs. 1, 3) as well as δ^13^C_head_ and δ^13^C_wing_ (p = 0.001). Tissue δ^13^C_abdomen_ and δ^13^C_wing_ values did show a significant difference (p = 0.68). Individual δ^15^N_wing_ values differed significantly from δ^15^N_abdomen_ (p < 0.001; Fig. 3b), and δ^15^N_head_ (p < 0.0001).

However, δ^15^N_abdomen_ was not significantly different from δ^15^N_head_ (p = 0.99; Fig. 2), indicating identical nitrogen sources for tissue synthesis. Overall, the slope of the relationship between δ^13^C_head_ and δ^15^N_head_ was -0.36 ± 0.01 (mean ± SE) (r^2^ = 0.47, F_[1,17]_ = 13.54, p= 0.002). The dataset of abdomen and wing isotopes samples analyzed were, however, hardly correlated (r^2^ = 0.02, p = 0.59 and r^2^ = 0.11, p = 0.19, respectively).

### Tissue specific stable isotope niche estimates

A summary of the carbon and nitrogen isotope niche metrics for pair-wise samples abdomen, head and wing is given in Table 3. Appendix Fig. 4a, b show the Bayesian estimates of the size corrected ellipse area (SEAc). The isotopic niche, measured as the standard ellipse area (SEA_C_), overlapped for all three tissues. However, the overlap was never complete but ranged between 14% (the lowest mean niche overlap between wing and head tissues) to 37% (the highest median niche area overlap was found between abdomen and head samples. The overlap between abdomen and wing was 27%. Abdominal tissues exhibited the largest SEA_C_ and wing tissues the smallest (estimated via Bayesian interference). The carbon isotope value of wing tissues had a significantly lower coefficient of variation than other tissues, presumably indicating a relatively narrow range of basal carbon resources utilized in the synthesis of this tissue. Relatively high nitrogen dietary divergence (CVn) was observed in the abdominal tissue sample, suggestive of tissue synthesis from dietary sources with high trophic diversity in terms of δ^15^N. Niche area estimates were much broader for abdominal tissues than the other two tissue types, in terms of both total area (TA) and standard ellipse area (SEA_C_) (Table 3). Abdominal SEA_C_ at tissue level was, on average, four times larger for abdomen than for wing and, and up to twice as large as for head samples. Appendix Fig. 4a shows the stable isotope bi-plots standard ellipse area illustrating the isotopic niche based on different tissue sections of *E. danica* (N = 17).

**Table 3:** Carbon and nitrogen isotope based niche metrics for abdomen head and wing of 17 individual insects

Tissue	SEA	SEAc	TA	CVc	CVn
Abdomen	1.47	1.57	4.0	3.96	7.78
Head	0.84	0.89	2.3	2.84	4.75
Wing	0.47	0.51	1.2	1.26	6.70

Shown are bayesian standard ellipse area (SEA%) and sample size corrected ellipse area (SEAc%), total area (TA%), coefficient of variation for carbon (CVc%) and nitrogen (CVn%) isotopes

## Discussion

Nutrient reserves acquired during the larval stages of *E. danica* that are not consumed during metamorphosis are allocated to somatic tissue maintenance and to re-structure the insect to the adult stage. Using dual isotope and triple-tissue analysis, we show a considerable metabolic nutrient routing and resource partitioning among somatic tissues. Results revealed that adult insect wing isotope values resemble those of detritus, indicating terrestrial nutrients as the most dominant energy sources during the aquatic larval stage of this speciesprior to emergence. This is not surprising, because its developing nymphal forms lives in burrows and feeds by filtering organic detritus [26]. Interestingly, however, this result demonstrates varying proportions of nutrient transfer originating from both terrestrial and aquatic sources to structural tissues maintenance, such as abdomen or head.

These findings support the hypothesis that insects depend on both autochthonous and allochthonous production energy supply for their activities. Clearly, the observed variation among tissue isotope values could be related to temporal shift in available dietary resources and their utilization during insect development [3, 26, 27]. Elsewhere, *E. danica* larvae have long been shown to exhibit a rapid response to environmental changes [5]. These findings imply a strong individual plasticity in nutrient allocation which might assist to maximize energy gain during growth, metamorphosis or starvations, and during rapid temporal resource fluctuations.

Results, therefore, emphasizes that tissue selection is important consideration for isotopic studies when using emerging arthropods. While assumptions of tissue selection are usually context-specific or context-dependent, our study indicate a careful tissue selection with in nutrient sources is essential in order avoid significant error, both in applying isotope signatures in estimating trophic level and in dietary proportions using mixing model approaches. Moreover, our isotopic model mainly assumes that all fractionation occurs during trophic transfer. However, some fractionation could also during metamorphosis. Particularly in isotopically less defined systems where dietary isotopic values are identical or exhibit higher variance, caution in tissue selection within sources is highly recommended.

In our study, the δ^13^C of terrestrial detritus is indistinguishable from sediment to seston δ^13^C values. This demonstrated that terrestrial detritus might be the major carbon sources associated with the large part of the organic matter in the food web, in general. Here, δ^13^C analyses are also suited for tracing lipid sources as lipids are depleted in δ^13^C relative to proteins, carbohydrates and whole tissues [28]. Thus, the relatively higher C/N mass ratio and lower δ^13^C exhibited in abdomen could be related to higher lipid content in this particular tissue. In contrast to the other two tissues, abdomen, incorporated the lowest percentage (57%) of terrestrial detritus.

In nutrient limited oligotrophic lakes, such as Lake Constance, these insects might be supplemented by allochthonous nutrient sources of lower nutritional value (i.e. higher C/N ratios) as this source might be more prolific over other autochthonous sources. The results and assumptions complement with previous studies that highlight the importance of allochthonous detritus as ann important energy source for profoundal macrozoobenthic fauna communities in the same Lake [29–31].

Finally, the δ^13^C and δ^15^N in abdomen and wing of insects were poorly correlated (unlike for head). Currently, mechanism responsible for the decoupling of carbon and nitrogen isotopes in food webs is not well understood, and we advocate a much broader sampling of both isotopes in the same tissues across taxa, across multiple instars, or across different time scales to resolve this issue and to increase the power of using these isotopes to track nutrient routing.

## Conclusions

Certainly terrestrial detritus (but also sediment organic matter) is the main nutrient source of mayfly, but its utilization as nutrient source, in terms of its carbon content may vary considerably over time and space, mainly due to seasonal and spatial differences in lake productivity. These findings indicate a case study as how ‘uncommon’ nutritional sources, such as periphyton and seston, play a lesser role in somatic resource synthesis in a model system of insects with burrowing larval stage. This also indicates that periphyton and seston based food availability and quality may be more important than previously assumed and may also have population-level consequences [32]. Future isotope-based studies should also assess the fatty acid and amino acid specific sources to individual maintenance. Generally, stable isotope profiling of emerging insects may prove an important method for monitoring the impacts of several processes including the effect of climate change on lake organic detritus and function as an early warning system of long-term shift in isotopic signals be detected.

In summary, considerable advances in the understanding of role of terrestrial and benthic production to aquatic food webs have been made through the use of stable isotopes [33]. Our results indicate how terrestrial and sediment, periphyton and seston based sources play a key yet intertwined nutritional role in emerging aquatic insects. In light of these findings, we note more investigation to be drawn to causes and consequences of differential nutrient routing strategies to somatic tissues demonstrated here. Indeed more research is required to obtain the isotopic discrimination values essential to refine the mixing model applied. Our study showed how isotopic differences between tissue types can retain significant variation in estimated ecological parameters, implying that tissue selection requires a crucial attention for isotopic studies when utilizing aquatic emerging insects [34]. Actual predictions made by isotopic mixing-models is difficult because of the general paucity of methods of sampling dietary sources consumed by individual and the basic power for the use of bulk isotopes to sufficiently reflect the pool of elements individuals assimilate [35]. The recent development of compound specific isotopes coupled to application of heavy labelling (isotopic tracers) could provide an important tool that can complement this isotope approach,

Nonetheless, sampling from more than 60 protected nature areas throughout Germany; researchers have recently reported a reduction in flying insect mass by about 76% over the last 27 years [36]. These results are drastic and future efforts on similar other surveys of specific aquatic insect species should be rewarding. Mayfly has long been used as bio-indicators of freshwater ecosystems and their nymph larval show rapid response to environmental changes. Therefore, developing a system to trace nutrient flow in this important species using isotopic tool will further help in conservation and management efforts in general.

## Figures and Tables

**Fig. 4:**
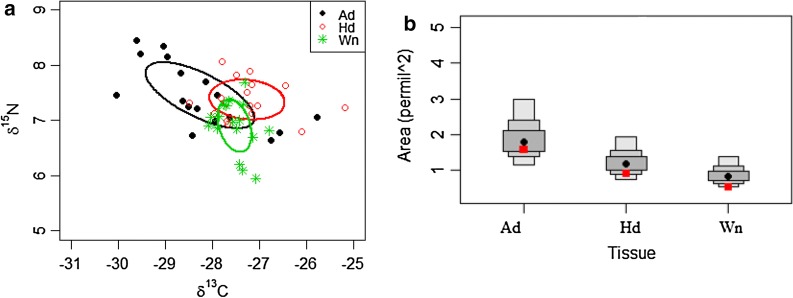
**a** Stable isotope bi-plots illustrating the isotopic niche based on different tissue sections of E*phemera danica.* The lines enclose the standard ellipse area (SEAc). **b** Bayesian estimates of the size corrected ellipse area (SEAc). Shaded density plots represent 50, 75 and 95% credible intervals in decreasing order of size, with SEA_B_ mode (standard ellipse area) indicated by a black circle and SEAc by a red square. *Ad* abdomen, *Hd* head and *Wn* wing

**Fig. 5:**
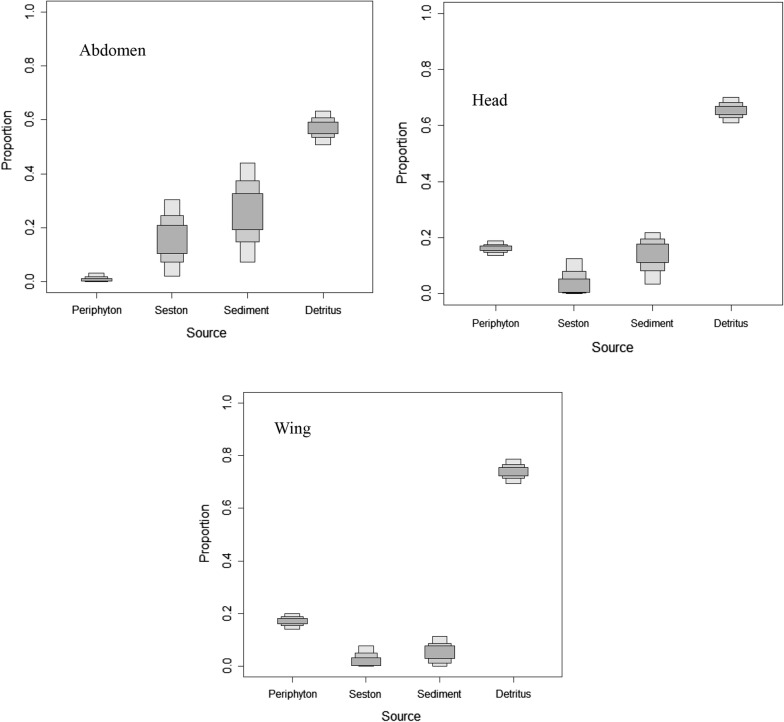
Boxplots show the proportional contribution (%) of different dietary sources to abdomen, head and wing

## Appendix

See Figures 4, 5.

## Abbreviations

Ad: abdomen
C: chemical symbol for carbon
CrI: credible intervals
CV: coefficient of variation
CVc: coefficient of variation for carbon
CVn: coefficient of variation for nitrogen
Hd: head
HSD: Tukey’s honest significant difference post hoc test
MCMC: Markov chain Monte Carlo
M: molar
MixSIAR: mixing model programmed in R
N: chemical symbol for nitrogen
PDB: Pee Dee Belemnite
SD: standard deviation
RM-ANOVA: repeated measure analysis of variance
SEA_B_: Bayesian estimates of ellipse area
SEA_C_: Bayesian estimates of the size corrected ellipse area
SIBER: stable isotope Bayesian ellipses
TA: total area
Wn: wing
δ^13^C: shift in the ^13^C/^12^C ratio of the sample relative to the reference standard (i.e. Pee Dee Belemnite)
δ^15^N: shift in the ^15^N/^14^N ratio of the sample relative to the reference standard (i.e. air)
